# Post-mortem CT: A Useful Tool to Confirm a Case of Suspected Sudden Cardiac Death

**DOI:** 10.7759/cureus.28021

**Published:** 2022-08-15

**Authors:** Karthi Vignesh Raj K, Abhishek Yadav, Gokul G, Amar Ranjan, Sudhir K Gupta

**Affiliations:** 1 Forensic Medicine, All India Institute of Medical Sciences, New Delhi, New Delhi, IND; 2 Forensic Medicine and Toxicology, All India Institute of Medical Sciences, New Delhi, New Delhi, IND; 3 Laboratory Oncology, All India Institute of Medical Sciences, New Delhi, New Delhi, IND

**Keywords:** post-mortem computed tomography (pmct), minimal/non-invasive autopsy, ventricle rupture, hemopericardium, sudden cardiac death

## Abstract

Sudden cardiac death (SCD) is defined as the unexpected death of an individual, not due to any extracardiac cause, occurring within one hour of symptom onset or within 24 hours of last being seen in good health if the death is unwitnessed. Forensic pathologists routinely encounter several SCD cases in their practice. The presentation of such cases can be of two types; firstly, with typical signs and symptoms suggestive of cardiac pathology, and secondly, devoid of any presentation history. This history helps forensic pathologists look for relevant findings during the autopsy examination. The authors intend to explore the feasibility of using advanced radiological techniques like post-mortem CT (PMCT) in determining the cause of death through a minimally invasive approach. In the present case, a 65-year-old male was found unresponsive at his residence on the morning of his death. He had a history of dull chest pain for the past two days, which had resolved after he self-prescribed a few medications. The presenting complaint of chest pain had started the intervening night prior to his death. The deceased was a known case of hypertension and was not compliant with treatment, as stated by the relatives. He was declared as brought dead by the treating emergency medicine physician at the Fortis Flt. Lt. Rajan Dhall Hospital and the body was sent by the authorities to the mortuary of the Department of Forensic Medicine, AIIMS, New Delhi for autopsy examination since an autopsy should be conducted by a government hospital or institute by law. PMCT depicted an alternate hyperdense and hypodense region circumferentially surrounding the heart, indicating hemopericardium. It was followed by a traditional autopsy and histopathology examination, which confirmed the presence of hemopericardium and left ventricular rupture associated with acute coronary insufficiency. Such cases with an indicative history, circumstantial evidence, and PMCT findings can be considered for minimal invasive autopsy. If the external findings indicate the application of physical force, then an explorative dissection could be done. Therefore, we conclude that PMCT can be used as a reliable tool for determining the cause of death in SCDs on a case-to-case basis.

## Introduction

Sudden cardiac death (SCD) is the unexpected death of an individual occurring within one hour of symptom onset or within 24 hours of last being seen in good health if the death is unwitnessed [1]. The time interval between the onset of symptoms and death is a major factor in determining whether a death is an SCD. This range for the time interval is not clearly defined as different authors have mentioned different intervals; however, it is one hour according to WHO guidelines. The common causes of SCD are ischemic heart diseases and non-ischemic cardiomyopathies. The ischemic causes are commonly attributed to atherosclerotic coronary artery disease (CAD). The different forms of CAD are coronary artery dissection, coronary artery thrombus, and vasculitis of the coronary artery [2]. Sudden death is a complication of acute myocardial infarction (AMI). Arrhythmias, cardiogenic shock, endocardial thrombus formation, ventricular rupture, aneurysm formation, papillary muscle dysfunction, and fibrinous epi- and pericarditis are known complications of myocardial infarction [3,4]. The complications arise due to poor perfusion and decreased oxygenation resulting in uncoordinated contractility of the ventricle and the atrium of the heart. On the other hand, the rupture of the walls of the heart occurs commonly due to myocardial infarction based on study findings [4]. SCD due to spontaneous cardiac rupture, which needs immediate intervention, mostly presents with chest pain [5-7]. In the present case, a holistic approach is used by including conventional autopsy, histopathology, and PMCT examination in a case who had died due to spontaneous left ventricular free wall rupture. In this report, we intend to explore the feasibility of using advanced radiological techniques like PMCT in determining the cause of death in cases with signs and symptoms suggestive of cardiac pathology and corroborating it with circumstantial evidence.

## Case presentation

A 65-year-old male was found unresponsive at his residence on the morning of his death. He had a history of dull chest pain for the past two days, which had resolved after he self-prescribed a few medications. The presenting complaint of chest pain had started the intervening night prior to his death. The deceased was a known case of hypertension and was non-compliant with treatment, as stated by the relatives. He was declared as brought dead by the treating emergency medicine physician at the Fortis Flt. Lt. Rajan Dhall Hospital and the body was sent by the authorities to the mortuary of the Department of Forensic Medicine, AIIMS, New Delhi for autopsy examination since an autopsy should be conducted by a government hospital or institute.

PMCT examination

**Figure 1:**
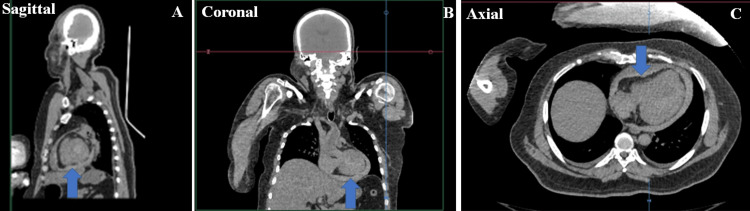
PMCT Images A, B, C: multiplanar reconstruction in PMCT showing the presence of hyperdense (white color) area separating the pericardium from the heart border suggestive of the presence of blood clots (blue arrow) PMCT: post-mortem computed tomography

Firstly, the body was subjected to PMCT scanning using a 16-slice Multi-Slice CT spiral scanner, Aquilion Lightning TSX-035A CT (Toshiba America Medical Systems, Tustin, CA). A 16 x 1-mm collimation was used for all the cases for data acquisition. All the raw data was processed into slices of 1-mm thickness. Multiplanar and 3D reconstruction were also done. The PMCT scanning protocol covered the entire body in three series: the first from the top of the head to the shoulders; the second from the neck, above the shoulders to the height of the pubic symphysis; and the third series covered the pelvis and lower limbs. The reconstructions were performed in the soft tissue, bone, and lung window, and the results were evaluated with the Vitrea software v.6.9.1. In PMCT, there was an alternate hyperdense and a hypodense region present concentrically inside the pericardium as visualized by multiplanar reconstruction techniques, indicating hemopericardium, and the same was discussed with a radiologist (Figures 1A, 1B, 1C).

Autopsy findings

**Figure 2:**
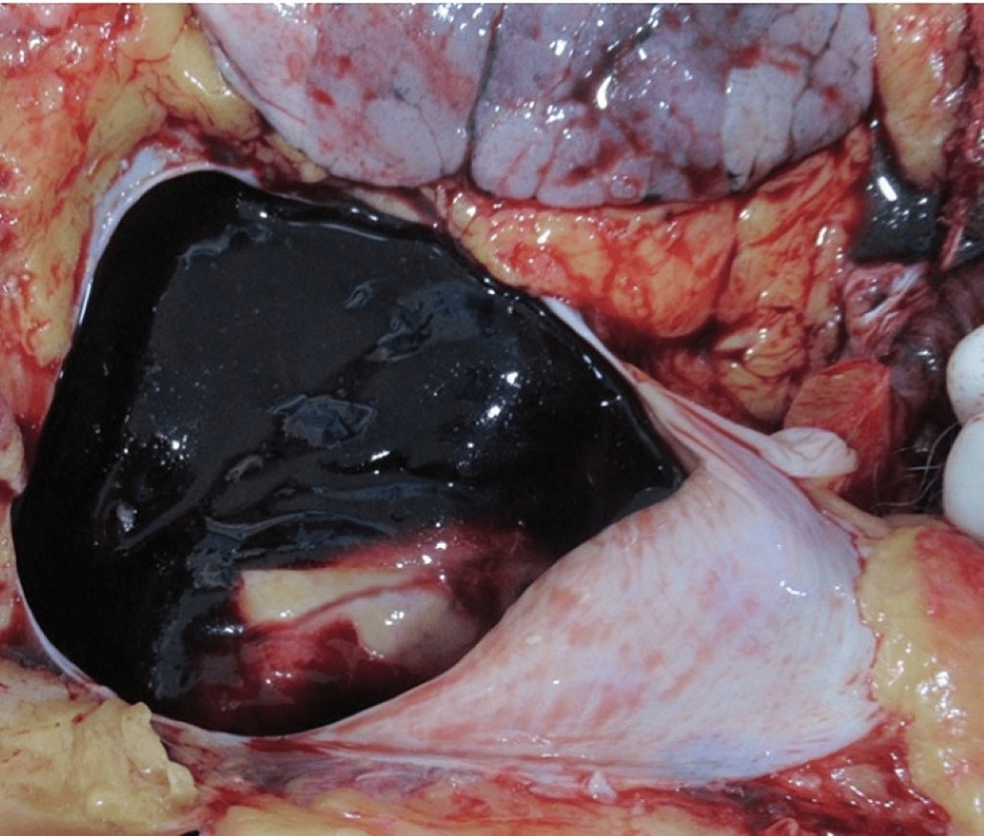
Hemopericardium Organized clotted blood present inside the pericardium

The deceased was of average build and moderately nourished. Rigor mortis was present all over the body. Lividity was present and fixed on the dependent parts of the body in a supine position with contact pallor. The face and upper parts of the chest were congested. Conjunctivae of both eyes were congested. Nailbeds of both hands had bluish discoloration. There were no external injuries anywhere on the body. The pericardial sac had bluish discoloration and bulging externally. On dissection, the pericardial cavity contained about 200 cc of clotted blood overlying the heart with liquid blood under the chambers posteriorly (Figure 2).

**Figure 3:**
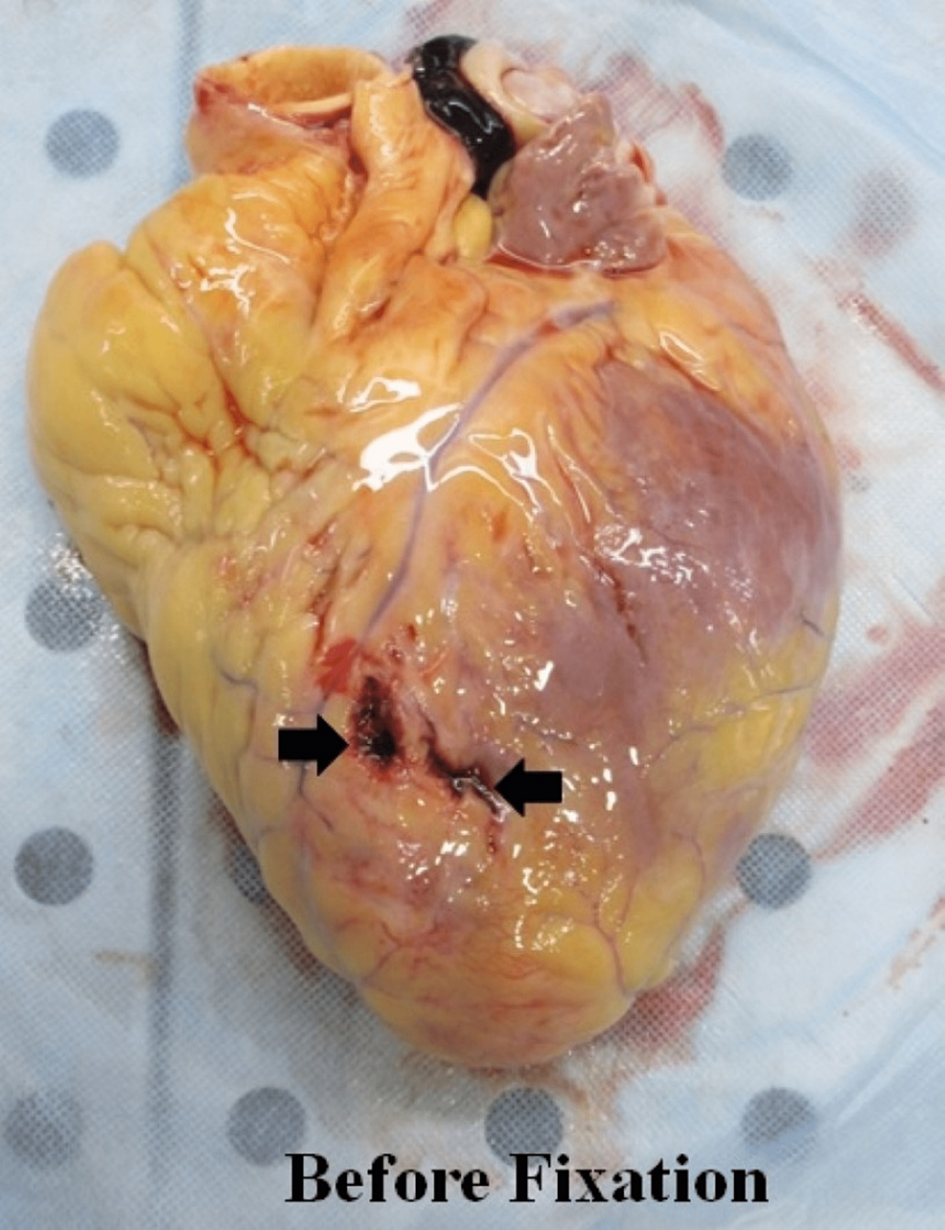
Rupture site before fixation Left ventricle rupture site on the anterior free wall before fixation

**Figure 4:**
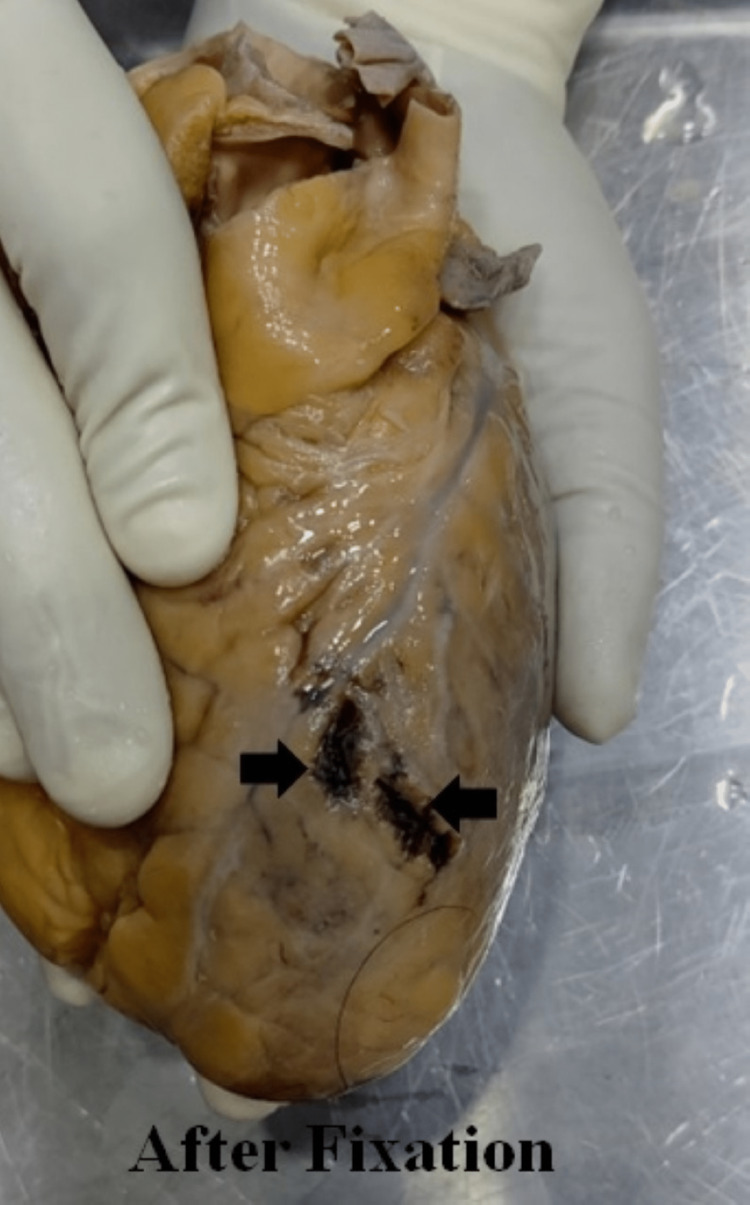
Rupture site after fixation Left ventricle rupture site on the anterior free wall after fixation

On cardiac examination, the anterior free wall of the left ventricle showed two small tears with irregular margins externally (Figure 3), measuring 1 cm x 1 cm x ventricle cavity deep, 1.8 cm x 1 cm x ventricle cavity deep adjacent to each other with an intervening intact fat layer. There were adherent clots at the rupture site, which were also seen post-fixation with formalin (Figure 4).

**Figure 5:**
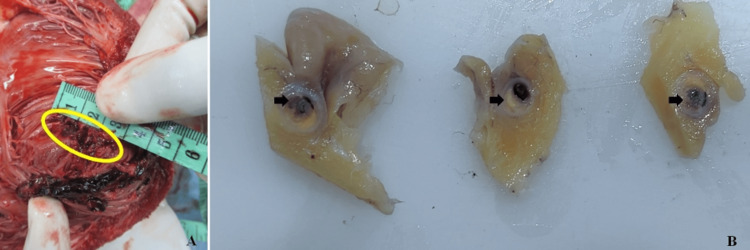
Rupture site and atherosclerotic occlusion A: rupture site in the myocardium of the left ventricle. B: serial sections of the left coronary artery after fixation showing complete stenosis

The internal examination of the chambers showed a through and through tear measuring 3 cm x 1 cm x ventricle cavity deep corresponding to the external tear and hemorrhagic infiltration in the surrounding myocardium (Figure 5A). The valves and walls of the remaining chambers were intact and unremarkable. The left coronary artery and its branches showed calcific atherosclerotic plaques with about 70-80% occlusion of the arterial lumen (Figure 5B).

Histopathological examination

**Figure 6:**
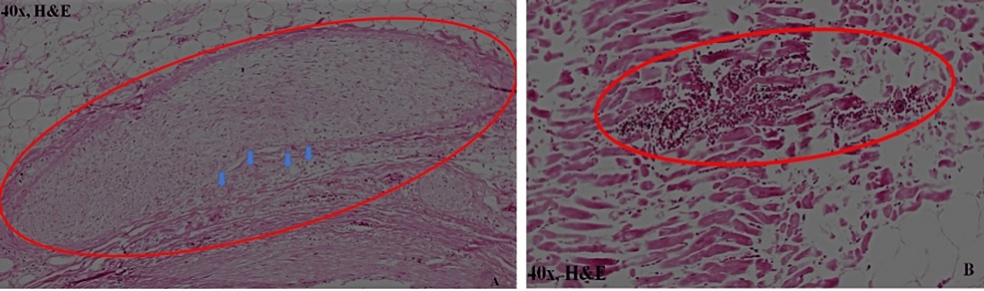
HP - atherosclerotic occlusion and rupture site A: proximal-most part of the left coronary artery showing intimal layer proliferation along with complete stenosis (red ellipse). Multiple foam cells are present (blue arrow). B: rupture of the myocardial fibers with necrosis and infiltration of neutrophils

**Figure 7:**
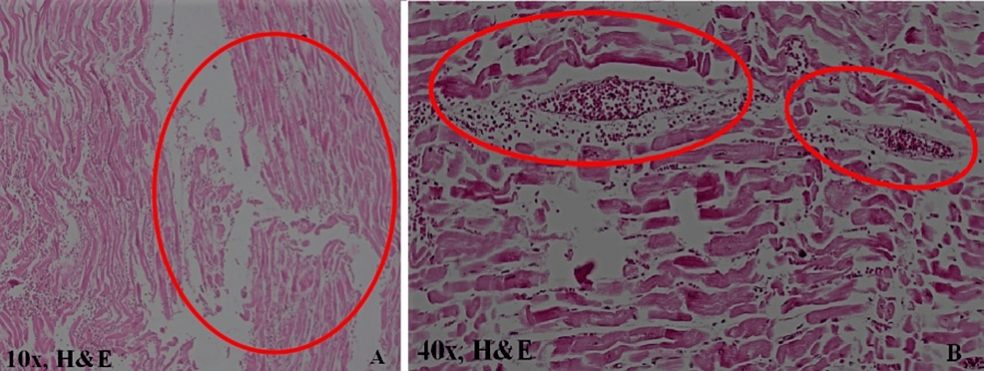
HP - rupture site A: the presence of the myocardial contraction bands. B: the presence of myocardium necrosis along with infiltration of neutrophils at the rupture site

The histopathology examination of the left coronary artery showed complete atherosclerotic occlusion with the presence of multiple foam cells. There was a proliferation of the intima and neutrophilic deposition (Figure 6A). The right coronary artery showed concentric atherosclerotic wall thickening with about 30% narrowing of the lumen. Both lungs were congested and oedematous. All other visceral organs showed evidence of chronic venous congestion. The histopathological examination of the heart showed rupture of the muscle fibers (Figures 6B, 7A) and necrosis at the rupture site on the left ventricle (Figure 7B). The cause of death was given as cardiac tamponade due to rupture of the left ventricular free wall with associated acute coronary insufficiency.

## Discussion

Sudden deaths are a major concern to forensic pathologists as there are high chances of foul play even though not all sudden deaths are homicides. Hence, such cases with clear circumstantial evidence and without any foul play should be considered and confirmed using PMCT to avoid unnecessary mutilation of the body. If the external findings raise suspicions about the application of external force, then explorative dissection must be done. Forensic pathologists encounter several SCD cases in their routine practice, which can be divided into two types; the first group presents with typical signs and symptoms suggestive of cardiac pathology, while the second group is devoid of any presentation history. This presentation history helps the forensic pathologists to look for relevant findings during the autopsy examination.

In 1647, William Harvey documented myocardial rupture due to sudden death, which was reported by Willis R in 1847 [8]. The term myocardial rupture denotes any acquired and complete defect in the muscular wall involving at least one chamber of the heart. It either occurs spontaneously due to several pathologies or following any traumatic event [9]. Krumbhaar and Crowell have stated that myocardial ruptures are very common in old age considering the age-related changes in the lumen of coronaries associated with causes like walking, eating, defecation, slight exertion, or even during sleep [10]. The frequent site of myocardial rupture is the left ventricular free wall associated with an acute infarct [11]. Yadav et al. have reported two pertinent cases; the first case was of an auto driver who collapsed due to chest pain in front of an eyewitness, while the deceased in the second case was a female who had chest pain and collapsed during sleep at night, which is a similar presentation to the current case [12]. DebBarma et al. have reported a case where death was sudden due to chest pain and happened at a cinema theatre [13]. The police investigation in the present case revealed that the deceased was a known case of hypertension, not on any regular medication, and with a history of chest pain for two days prior to his death. The similarity, in all cases discussed, pertains to the presentation prior to the death, which was chest pain and sudden collapse.

In cases with a history of signs and symptoms suggestive of cardiac pathology, is it really necessary to perform a complete invasive autopsy if an institute is equipped with a CT machine? To address this question, the following points were considered. After performing a proper external examination, the PMCT was conducted, which also showed cardiac tamponade due to rupture of the ventricle and calcification of the coronary artery. The forensic pathologist, while confirming the cause of death using PMCT, also needs to consider the cardiac rupture caused due to cardiopulmonary resuscitation (CPR) as reported in various studies in the literature [14-18]. Firstly, this can be confirmed from the details mentioned in the treatment records prior to death. Then, the absence of rib fractures overlying the heart along with the second-fifth rib along the parasternal, midclavicular line, and anterior axillary line in PMCT would rule out CPR-associated heart rupture.

The vitality can be confirmed by visualizing the pattern of blood collected inside the pericardium on PMCT from the two distinct patterns as reported in the literature [19,20]. A horizontal layering of density that mimics when blood products separate in the great vessels during normal hypostasis is one type. The second pattern is the hyperdense ring appearance, which has higher significance in this anatomic location to indicate the vitality of rupture. In this pattern, a concentric ring of high density immediately surrounds the heart, which is due to the clot, and a ring of low density overlies the hyperdensity region due to the serum. This implies that the hemorrhage had occurred when the heart was still beating, i.e., during the antemortem period as seen in this case. Thus, we express the view that avoiding the internal dissection in SCDs due to rupture of ventricles can be considered on a case-to-case basis and PMCT can be considered a reliable tool.

## Conclusions

Based on the comparison between PMCT and conventional autopsy independently, each methodology involves consideration of the circumstantial evidence and findings submitted by the investigating officer and a proper external examination. Thus, in the place of internal dissection, PMCT scanning can be considered a holistic approach to find the cause of death in cases with an obvious history and without any suspicion of foul play. In addition, it addresses the most important concept of humanitarian forensic medicine. Since there is obvious evidence that the deceased died in the presence of relatives and also that foul play was ruled out by the investigating officers, the need for explorative dissection is minimal in these types of cases. Therefore, PMCT could serve the purpose of finding the cause of death in SCDs due to ventricular rupture, thereby maintaining the dignity of the dead.
